# Inter-Hemispheric Coordination and Ageing in Visual Working Memory: A Distributed Framework

**DOI:** 10.3390/brainsci16060641

**Published:** 2026-06-16

**Authors:** Jean-François Delvenne

**Affiliations:** School of Psychology, Faculty of Medicine and Health, University of Leeds, Leeds LS2 9JT, UK; j.f.delvenne@leeds.ac.uk

**Keywords:** visual working memory, ageing, inter-hemispheric communication, corpus callosum, divided visual field, coordination, attention, binding

## Abstract

**Highlights:**

**What are the main findings?**

- Ageing affects multiple visual working memory processes, including precision, storage, binding, and attentional control.
- The paper argues that reduced inter-hemispheric communication may underlie several of these deficits.

**What are the implications of the main findings?**

- The review proposes inter-hemispheric coordination as a unifying framework for visual working memory ageing.
- Understanding hemispheric coordination may help refine models of cognitive ageing.

**Abstract:**

Visual working memory (VWM) declines with age and has been explained by multiple mechanisms, including reduced precision, capacity limitations, binding deficits, and altered attentional control. However, these accounts are typically framed within a unitary processing architecture and do not fully capture the distributed nature of visual cognition. This review advances a coordination-based framework in which age-related differences in VWM are understood as partly reflecting reduced efficiency in integrating and regulating representations across the two cerebral hemispheres. Behavioural, electrophysiological, and neurophysiological evidence is synthesised to characterise the role of inter-hemispheric communication in VWM. Age-related changes in corpus callosum structure and function are then considered in relation to these coordination processes. Deficits in precision, capacity, binding, and attention are proposed to reflect different behavioural expressions of a common limitation in coordinating distributed representations, providing a unifying account of multiple behavioural signatures, particularly under conditions that place high demands on inter-hemispheric coordination. The framework offers a mechanistic explanation of the task-dependent nature of ageing effects and generates testable predictions for future research, highlighting the role of network-level coordination mechanisms in cognitive ageing.

## 1. Introduction

Visual working memory (VWM), the capacity to temporarily maintain and manipulate visual information, is critical for everyday tasks like searching, navigation, and driving. A robust finding across cognitive ageing research is that VWM performance declines with advancing age [1]. While this decline is well established, its underlying mechanisms remain debated. Rather than reflecting a unitary deficit, accumulating evidence indicates that age-related differences in VWM arise from multiple interacting factors that affect the selection, representation, and control of visual information.

One prominent line of work emphasises a decline in the precision of memory representations, with older adults storing items less precisely, resulting in noisier recall and greater response variability [2,3,4,5]. Within this framework, VWM decline reflects a degradation in representational fidelity rather than a strict reduction in the number of stored items. Closely related accounts focus on capacity and load sensitivity, proposing that both representational and active-maintenance systems become less efficient with age. As task demands increase, performance in older adults deteriorates more steeply, suggesting a reduced ability to sustain multiple representations simultaneously [1,6,7,8]. Together, these findings indicate that ageing affects both the quality and scalability of VWM representations.

A second line of research highlights deficits in binding and relational processing, which are essential for maintaining coherent object representations. Some studies report impairments in binding items to their spatial or contextual attributes, whereas binding of surface features often appears relatively preserved [9,10,11]. However, this pattern is not uniform. Forsberg et al. argue that apparent binding deficits may partly reflect strategic adaptations, such as increased reliance on verbal rehearsal, rather than a fundamental impairment in binding mechanisms [12]. This suggests that age-related differences in binding may emerge most clearly under conditions that limit compensatory strategies or increase demands on the flexible use of relational information.

A third cluster of accounts attributes VWM decline to changes in attentional control and inhibitory processes. Older adults show reduced efficiency in selecting relevant information and suppressing irrelevant inputs, leading to greater susceptibility to distraction and increased intrusion from non-target items [13,14,15,16,17,18]. In addition, ageing has been associated with a shift from proactive to more reactive control strategies, limiting the ability to prevent interference before it arises [19]. Whereas proactive control involves the anticipatory maintenance of task goals to minimise interference, reactive control relies on resolving interference after it has occurred. These deficits are likely to have cascading effects across processing stages, as inefficient selection at encoding increases the likelihood of representational overlap and competition at later stages.

Importantly, these mechanisms are not independent. Reduced precision increases the overlap between representations; inefficient attentional control allows irrelevant information to enter and compete for limited resources; and binding deficits reflect difficulties in maintaining integrated representations under conditions of interference or high demand. Capacity limitations, in turn, become more pronounced as these inefficiencies accumulate, particularly under high-load conditions.

Across these accounts, a common theme emerges: age-related decline in VWM reflects a reduced ability to coordinate multiple representations and processes under constraint. From this perspective, VWM decline may be more parsimoniously characterised as a deficit in the coordination of information across representations, processes, and task demands. This integrative view provides a unifying framework for disparate findings and generates the prediction that age differences should be most pronounced in tasks that place high demands on the integration, segregation, and control of multiple representations. It also provides a principled bridge to mechanisms that critically depend on the coordination of distributed neural systems, including inter-hemispheric coordination. Throughout this review, the term inter-hemispheric coordination is used to refer not simply to structural connectivity, but to the dynamic processes by which representations are transferred, aligned in time, selectively accessed, and protected from interference across the two hemispheres. This definition emphasises coordination as an active, control-dependent mechanism rather than a passive communication channel.

Visual cognition is fundamentally distributed across the two cerebral hemispheres, with each hemisphere primarily processing information from the contralateral visual field. As a consequence, many VWM representations, particularly those involving spatially distributed stimuli, are initially encoded in a lateralised manner. Maintaining and using such representations therefore requires coordination between hemispheres, mediated by inter-hemispheric communication pathways, most notably the corpus callosum (CC). Notably, not all VWM tasks place equivalent demands on such coordination: tasks dominated by local encoding or storage processes may rely primarily on intra-hemispheric mechanisms, whereas tasks requiring integration, comparison, or selective access across spatially distributed representations place greater demands on inter-hemispheric coordination.

Although the CC represents the principal anatomical pathway linking the two hemispheres and therefore constitutes a central focus of the present review, inter-hemispheric coordination is likely to depend on a broader network of cortical and subcortical structures. In particular, the pulvinar has been implicated in attentional coordination, neural synchronisation, and the routing of information across distributed cortical regions [20,21]. These mechanisms may complement callosal communication by regulating the timing and prioritisation of information exchange across large-scale neural systems. Nevertheless, because the CC has been most extensively investigated in relation to hemispheric communication and cognitive ageing, the present review focuses primarily on its role in supporting inter-hemispheric coordination in VWM.

Nevertheless, the role of inter-hemispheric coordination in VWM has received relatively little attention in the cognitive ageing literature. Although a small number of studies have examined age-related changes in inter-hemispheric processing and callosal function [22,23,24], most theoretical accounts of VWM ageing have focused on local mechanisms such as storage limitations, binding deficits, or attentional control. Yet, ageing is associated with substantial alterations in the structure and function of the CC, including reductions in white matter integrity and changes in functional connectivity patterns [25,26,27,28,29]. These changes are likely to influence the coordination of distributed representations across hemispheres.

Inter-hemispheric processing has been investigated using a range of behavioural and neurophysiological approaches. Behavioural studies have frequently employed divided visual field paradigms, which manipulate whether information is processed within or across hemispheres, whereas electrophysiological and neuroimaging methods have examined the neural mechanisms supporting inter-hemispheric coordination, including oscillatory synchronisation, functional connectivity, and callosal integrity. Together, these approaches provide complementary insights into how information is integrated and regulated across the two hemispheres.

The present review advances the proposal that age-related differences in VWM cannot be fully understood without considering inter-hemispheric coordination. Specifically, it suggests that some deficits traditionally attributed to reduced capacity, precision, binding, or attentional control may also reflect a diminished ability to integrate and coordinate information across hemispheres, particularly under conditions that require distributed processing. This perspective provides a coherent account of the well-documented task dependence of ageing effects, whereby deficits are more pronounced in tasks that impose higher demands on integration, comparison, or attentional coordination [6]. The present review builds on behavioural and neurophysiological findings showing that inter-hemispheric interactions in VWM depend on attentional selection, task demands, and coordination efficiency. Age-related decline in VWM is proposed to partly reflect a disruption of this coordination, alongside changes in storage capacity and precision.

The present article is a narrative review intended to integrate evidence across cognitive ageing, visual working memory, and inter-hemispheric coordination. Relevant studies were identified through the authors’ knowledge of the literature and targeted searches of major databases (e.g., PsycINFO, PubMed, and Web of Science). The aim was not to provide a systematic review of all available evidence, but rather to synthesise key findings and develop a theoretical framework linking age-related changes in inter-hemispheric coordination to VWM performance.

The argument developed in this review proceeds in four stages. First, the hemispheric organisation of visual processing and its implications for VWM are outlined. Second, behavioural and neurophysiological evidence on inter-hemispheric coordination in VWM is synthesised. Third, the effects of ageing on the structure and function of inter-hemispheric pathways are examined, together with the consequences of these changes for VWM performance. Finally, these findings are integrated into a coordination-based framework of VWM ageing, from which implications for theory and future research are derived. This framework explicitly conceptualises age-related changes in VWM in terms of inter-hemispheric coordination processes.

## 2. Hemispheric Organisation of Visual Working Memory

### 2.1. Contralateral Organisation

The visual system is organised in a fundamentally contralateral manner, with inputs from each visual field projecting to the opposite hemisphere [30]. This organisation extends beyond the early visual cortex into higher-order regions implicated in attention and working memory. Behavioural, electrophysiological, and neuroimaging studies have demonstrated that VWM representations are initially encoded in a lateralised fashion, reflecting the spatial location of stimuli [31,32,33,34].

One of the most widely used neural markers of VWM, the contralateral delay activity (CDA), provides compelling evidence for this lateralised organisation. The CDA is a sustained posterior negativity observed contralateral to attended items and typically scales with the number of representations maintained in memory [34]. Although originally interpreted primarily as an index of storage capacity, subsequent work suggests that the CDA is also influenced by attentional selection, filtering efficiency, and the prioritisation of information within VWM [33,35]. As such, the CDA may reflect the interaction between memory maintenance and attentional control processes rather than storage alone. Nevertheless, its lateralised nature provides important evidence that VWM representations are initially organised in a hemisphere-specific manner.

However, despite this contralateral organisation, electrophysiological evidence indicates that VWM capacity reflects a shared resource across hemifields rather than independent hemispheric stores. Delvenne et al. showed that, although the CDA is measured contralaterally, its amplitude tracks the total number of items maintained across both hemifields, not only those in the contralateral field [36]. When items were presented within a single hemifield, CDA amplitude increased up to approximately four items, indicating capacity limits. When items were distributed across both hemifields, the CDA reached the same asymptote with only two items per hemifield (approximately four items in total). This pattern suggests that capacity is shared across hemispheres and that limits arise at the level of integrated processing. Because information is often distributed across visual fields, the effective use of these representations depends on inter-hemispheric coordination.

### 2.2. Hemispheric Asymmetries

In addition to contralateral organisation, the two hemispheres exhibit functional asymmetries that shape VWM processing. The right hemisphere is typically associated with spatial and global aspects of visual processing, whereas the left hemisphere is more specialised for local and feature-based information [37,38,39]. These asymmetries extend to VWM, with spatial maintenance showing a strong right-hemisphere bias and object/feature-based representations showing a relative left-hemisphere bias [40,41].

Crucially, hemispheric asymmetries vary with feature load. At the neural level, the left intraparietal sulcus (IPS), whose activity tracks the number of items maintained in VWM [42,43], primarily represents contralateral information, whereas the right IPS represents information across both visual fields [44]. Behaviourally, this is reflected in a left visual field advantage for simple, single-feature stimuli, and a right visual field advantage when objects contain multiple features [45], consistent with bilateral support for representations in the right hemifield.

Taken together, these asymmetries (i.e., right-lateralised support for spatial/global processing, left-lateralised support for feature-based representations, and a more bilateral coding profile in right parietal cortex) indicate that VWM relies on complementary, hemisphere-specific computations. When task demands combine these computations (e.g., integrating global spatial structure with local feature detail or operating under higher feature load), effective performance depends on inter-hemispheric coordination, rather than on processing within a single hemisphere.

This perspective aligns with a broader shift in cognitive neuroscience from viewing the hemispheres as relatively independent systems to recognising their functional interdependence [46,47]. In the context of VWM, this implies that performance depends not only on hemisphere-specific processing, but also on the efficiency with which information is exchanged and integrated across hemispheres, particularly under conditions of increased load or complexity.

A key question is how these lateralised and asymmetric processes interact to support coherent visual working memory. While Section 2.1 and Section 2.2 highlight the distributed and specialised nature of VWM representations, they also imply that effective performance requires coordination across hemispheres when information is spatially distributed or computationally complex. The following section therefore examines the mechanisms of inter-hemispheric communication in VWM and how behavioural and neurophysiological evidence from divided visual field paradigms has been used to characterise the balance between hemispheric independence and integration.

## 3. Inter-Hemispheric Communication in Visual Working Memory

Building on this hemispheric organisation, divided visual field (DVF) paradigms provide key evidence for how inter-hemispheric coordination supports VWM performance. Presenting items bilaterally across hemifields enhances VWM capacity [8,48,49,50] and improves precision [49,51] compared with presenting the same number of items within a single hemifield, an effect known as the bilateral field advantage (BFA).

Further work indicates that the BFA reflects a balance between partially independent hemispheric resources and their joint maintenance. As discussed in the previous section, electrophysiological evidence indicates that, although each hemisphere contributes to storage, capacity limits arise at the level of integrated processing across hemifields [36]. Behavioural evidence similarly shows that performance in bilateral conditions does not reach the simple sum of both hemispheres’ capacities, implying a shared active maintenance system that must coordinate across hemispheres [8]. In this sense, overall capacity reflects both hemisphere-specific resources and the efficiency of their joint maintenance.

Importantly, the BFA is not observed uniformly across all paradigms and appears to depend on task demands. For example, Chakravarthi and Cavanagh found a bilateral advantage when targets had to be selected under crowded conditions, but not when targets were isolated, suggesting that the effect depends on the need to resolve competition among stimuli [52]. Similarly, previous work indicates that the BFA is less consistent for colour or identity information than for spatial or orientation-based memory and may depend on the specific task procedure used [48,50]. Moreover, the magnitude of the advantage is typically smaller than would be predicted by a strict hemispheric-independence account, suggesting that processing relies on interactions between hemisphere-specific resources and shared maintenance mechanisms rather than entirely separate stores [8,36,50]. When attention is actively engaged, through cues or the need to resist distractors, selection and maintenance operate more effectively when relevant items are distributed across hemifields [53,54,55]. Thus, the BFA is not a simple sensory effect, but reflects the dynamic coordination of hemispheric resources, whose contribution varies according to stimulus domain, task requirements, and attentional demands.

Neurophysiological evidence further links these behavioural effects to inter-hemispheric coordination mechanisms. Sattelberger et al. showed that bilateral advantages are associated with dynamic changes in alpha and alpha–beta oscillations and increased interareal synchronisation, reflecting coordinated neural activity between bilateral visual cortices, parietal cortex, and prefrontal cortex [49]. In particular, higher inter-hemispheric phase synchronisation scales with memory load and predicts greater capacity, faster responses, and higher precision in bilateral relative to unilateral conditions, providing direct evidence that coordinated neural activity across hemispheres underpins improved VWM performance.

Converging neurophysiological evidence suggests that inter-hemispheric communication is an active process rather than a passive relay. For example, using electrophysiological recordings in non-human primates, Brincat et al. reported changes in the neural coding of working memory representations when remembered information shifted between visual hemifields [56]. Although the study did not directly measure callosal communication or ageing-related processes and cannot determine whether information was actively transferred between hemispheres, the findings are consistent with the possibility that working memory representations may be reconfigured as they become represented within a different hemispheric network. In practical terms, this suggests that information may not always be maintained in an identical format across distributed neural systems. More broadly, these findings support the view that effective inter-hemispheric coordination may involve more than the simple exchange of information, potentially requiring the alignment and transformation of representations across hemispheric networks.

Taken together, these findings support a hybrid view of VWM organisation. Processing is partly lateralised, allowing each hemisphere to contribute relatively independently under some conditions; however, effective performance ultimately depends on the coordination of distributed representations.

This framework has important implications for understanding variability in VWM, including age-related changes. If communication across hemispheres is required for integrating distributed information, then reductions in the efficiency of this communication, such as those associated with structural and functional changes in the CC, would be expected to place particular demands on tasks that rely on cross-hemispheric coordination. In this sense, VWM limitations may arise not only from local storage constraints but also from the efficiency with which information is coordinated across the two hemispheres. From this perspective, age-related decline would be expected to disproportionately affect tasks that place high demands on inter-hemispheric coordination, such as those requiring integration or comparison across hemifields, while leaving more localised processing relatively preserved. More generally, this framework predicts that ageing-related variability in VWM performance should scale with the extent to which tasks depend on the dynamic transfer, alignment, and control of distributed representations across hemispheres.

Building on this evidence, the next section examines how ageing affects inter-hemispheric communication and the consequences of these changes for VWM performance. Particular emphasis is placed on age-related alterations in CC structure and functional connectivity, and on how reductions in coordination efficiency may impair the integration and regulation of distributed representations across hemispheres.

## 4. Ageing and Inter-Hemispheric Communication in Visual Working Memory

Ageing is associated with widespread changes in brain structure and function, with particularly pronounced effects on white matter pathways, including the CC, the principal anatomical connection between the two hemispheres. Evidence from structural MRI indicates age-related macrostructural changes in the CC, including reductions in callosal area and volume, whereas diffusion-based studies reveal declines in white matter microstructural integrity, reflected in measures such as reduced fractional anisotropy and increased diffusivity [26,28,29]. These alterations are not uniform: anterior regions (e.g., genu) typically show earlier and greater decline than posterior regions (e.g., splenium) [57], although posterior pathways are also affected at later stages of ageing [58].

Both macrostructural and microstructural changes are likely to have functional consequences for the coordination of distributed neural activity [25,59]. Consistent with this view, ageing is associated with altered functional connectivity, including reduced inter-hemispheric synchronisation [60,61] and, in some cases, increased bilateral activation [62,63]. One influential interpretation of this pattern is provided by the HAROLD model [64], which characterises ageing in terms of reduced hemispheric asymmetry and increased bilateral recruitment.

However, increased bilateral activation does not necessarily reflect more efficient inter-hemispheric coordination and may instead indicate compensatory recruitment in response to declining neural resources. Importantly, compensatory and coordination-based accounts make different predictions. Compensation models, such as HAROLD [64], propose that bilateral recruitment helps maintain performance by engaging additional neural resources and therefore predict a positive relationship between bilateral activation and task performance. In contrast, a coordination-based account predicts that increased bilateral activation may sometimes reflect reduced efficiency in integrating and regulating distributed processing, particularly when inter-hemispheric communication is compromised. Consistent with this interpretation, Tagliabue et al. showed that, compared with younger adults, older adults exhibited a shift in processing during visuo-spatial working memory tasks: younger adults relied more on specialised, lateralised processing within a single hemisphere, whereas older adults engaged both hemispheres more simultaneously [65]. This increased bilateral engagement may therefore reflect a greater reliance on distributed processing across hemispheres, either as a compensatory response or as a consequence of reduced processing specialisation. Distinguishing between these possibilities will require examining the relationships among bilateral recruitment, behavioural performance, and measures of inter-hemispheric connectivity.

Crucially, structural integrity supports performance but does not fully account for age-related decline. For example, a larger anterior and mid-body CC area in older adults is associated with better working memory and processing speed, yet performance remains below that of younger adults even when CC size is comparable [24]. This indicates that an intact structure is beneficial but not sufficient for optimal coordination.

Within VWM, these changes point to a specific vulnerability of inter-hemispheric coordination, particularly in tasks requiring comparison or integration across hemifields. For example, using a divided visual field one-back task, Delvenne showed that younger adults performed similarly when successive stimuli were presented within the same or across opposite hemifields, whereas older adults exhibited a marked decline when matching required cross-hemifield integration [23]. Given the contralateral organisation of visual memory representations, matching across hemifields necessarily requires inter-hemispheric coordination. The selective impairment observed in older adults is therefore consistent with reduced efficiency in transferring and integrating information across hemispheres, rather than a general reduction in VWM capacity.

Converging evidence from individual differences supports this interpretation. In a VWM task requiring attention to be split across hemifields, individuals with lower callosal connectivity show increased rates of illusory conjunctions—errors in which features from different items are incorrectly combined—particularly when information must be integrated across visual hemifields [66]. This pattern indicates that reduced inter-hemispheric connectivity is associated not only with impaired transfer but also with degraded cross-hemispheric feature integration, leading to less precise representations.

Importantly, inter-hemispheric communication can both facilitate integration and propagate interference. In a divided-field Stroop task, in which target and distracting information were presented either within the same visual hemifield or across opposite hemifields, older adults showed reduced susceptibility to interference when target and distractor processing involved opposite rather than the same hemisphere, whereas younger adults were similarly susceptible in both conditions [22]. This pattern suggests that communication across hemispheres can facilitate integration but can also propagate interference between competing representations. This further supports the view that coordination is a dynamic process whose effects depend on task demands rather than a uniformly beneficial mechanism.

Overall, the evidence indicates that age-related decline in VWM partly reflects a disruption of inter-hemispheric coordination. Rather than being attributable solely to local processing deficits, performance limitations appear to arise from constraints on the integration and regulation of distributed representations, consistent with the communication framework outlined in Section 3. This account predicts that deficits will be most pronounced in tasks requiring integration, comparison, or coordinated selection across hemispheres, thereby providing a mechanistic link between age-related changes in the CC and behavioural limitations in VWM.

## 5. Towards a Coordination-Based Account of VWM Ageing

### 5.1. Integrating Existing Accounts

**Figure 1:**
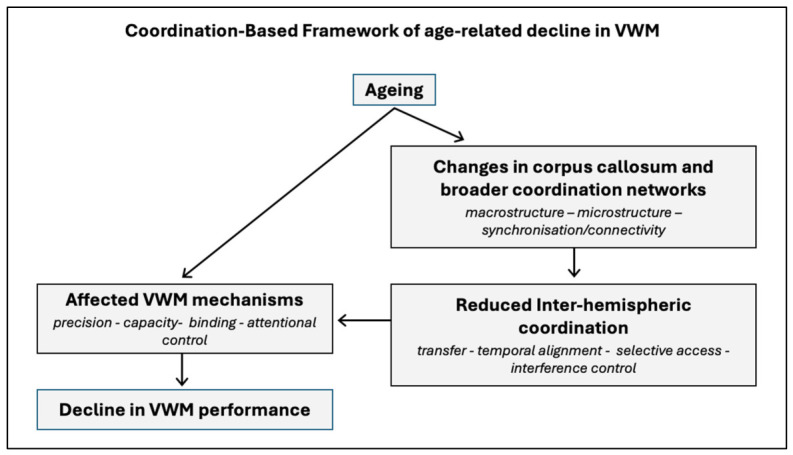
Coordination-based framework of age-related decline in VWM. Ageing is proposed to affect VWM through two complementary pathways. First, ageing directly influences core VWM mechanisms, including representational precision, storage capacity, binding, and attentional control. Second, age-related changes in the CC and broader coordination networks reduce the efficiency of inter-hemispheric coordination, encompassing processes such as transfer, temporal alignment, selective access, and interference control. Reduced inter-hemispheric coordination may further contribute to impairments in core VWM mechanisms, particularly under conditions requiring the integration, comparison, or selection of information across visual hemifields. Together, these processes contribute to age-related decline in VWM performance.

The preceding sections suggest that age-related differences in VWM are unlikely to reflect a single underlying deficit. Instead, mechanisms involving precision, capacity, binding, and attentional control appear to capture different aspects of a broader coordination constraint. From this perspective, performance depends not only on local processing efficiency but also on the ability to integrate, align, and regulate distributed representations across hemispheres (Figure 1). Unlike accounts that interpret bilateral recruitment primarily as compensatory resource upregulation, the present framework emphasises the efficiency and fidelity of coordination processes themselves. It is also distinct from general slowing and global neural noise accounts, which propose diffuse reductions in processing speed or signal quality; here, the critical limitation lies in the dynamic coordination of information across hemispheres.

Within this framework, apparent reductions in representational precision [2,3,4,5] can be interpreted as a consequence of disrupted coordination across distributed neural codes. When representations must be coordinated across hemispheres, maintaining high fidelity depends on temporal alignment and synchronisation across regions [67]. Computational accounts further support this view, showing that age-related changes in underlying neural dynamics can give rise to apparent declines in precision and capacity without invoking distinct mechanisms [2]. Age-related reductions in synchrony or increases in neural noise may therefore manifest as reduced precision, particularly in tasks requiring cross-hemifield comparison or integration.

A similar reinterpretation applies to capacity limitations [1,6,7,8]. Rather than reflecting a fixed storage limit, capacity can be understood as emerging from the demands placed on coordination mechanisms. As the number of items increases, demands on coordination also increase. Age-related declines in performance under high load may therefore reflect a reduced ability to sustain coherent multi-item representations across distributed systems, rather than a simple reduction in storage capacity.

Findings traditionally attributed to binding deficits [9,10,11] can also be recast within this framework. Binding depends on integrating features and contextual information into coherent representations, a process that becomes more demanding when information is distributed across hemispheres. Consistent with the evidence reviewed in Section 4, age-related reductions in callosal connectivity may contribute to increased illusory conjunctions under cross-hemifield conditions [66], indicating impaired integration of distributed features. Crucially, this account distinguishes between local binding processes, which may remain relatively intact, and distributed binding, which places greater demands on inter-hemispheric coordination.

Finally, changes in attentional control and inhibition [13,14,15,16,17,18] can be understood as contributing to coordination failure. Efficient selection and suppression are necessary for regulating activity across hemispheres, particularly when relevant and irrelevant information are distributed across hemifields. Age-related reductions in top-down control [68] may therefore increase interference between representations, amplifying coordination demands rather than acting as an independent source of decline.

Taken together, these mechanisms may not reflect distinct underlying deficits but rather different behavioural expressions of a common limitation in coordinating distributed representations. This framework predicts that age-related deficits will be minimal in tasks relying primarily on local processing and amplified in tasks requiring integration, comparison, or controlled selection across hemispheres. Although direct evidence linking coordination to each mechanism remains limited, this account is consistent with converging behavioural, neurophysiological, and connectivity-based findings.

More broadly, the coordination-based framework provides a unifying link between behavioural patterns and underlying neural architecture by emphasising the efficiency with which information is coordinated across hemispheres. In this view, age-related differences are inherently task-dependent because they emerge most clearly when coordination demands are high, offering a concise mechanistic explanation without invoking multiple independent deficits.

### 5.2. Limitations and Boundary Conditions

The present framework is not intended to explain all forms of age-related decline in VWM. Tasks relying primarily on local encoding, maintenance, or retrieval processes may be relatively insensitive to inter-hemispheric coordination demands. Under such conditions, age-related differences may be better explained by local representational degradation, attentional limitations, or general reductions in processing efficiency.

A second limitation is that direct evidence linking inter-hemispheric coordination to each proposed mechanism remains relatively sparse. Much of the argument developed here is based on converging evidence drawn from separate studies on VWM, ageing, corpus callosum integrity, and inter-hemispheric communication. Future studies that simultaneously assess these factors will be required to establish more direct causal relationships.

Furthermore, the framework should be viewed as complementary to, rather than replacing, existing accounts of ageing. General slowing, neural noise, and compensatory recruitment may themselves contribute to coordination failures, and future work will be needed to clarify how these mechanisms interact.

## 6. Future Directions

The coordination-based framework outlined in this review opens several avenues for future research, both theoretical and empirical. These directions follow from the proposal that age-related decline in VWM reflects limitations in coordinating distributed representations and therefore prioritise approaches that isolate and quantify the contribution of inter-hemispheric coordination to VWM performance. In particular, this framework generates specific predictions about how coordination demands should modulate age-related differences in VWM performance, some of which already receive preliminary support from the existing literature reviewed above.

### 6.1. Linking VWM Performance to Callosal Integrity

A key priority is to establish more direct links between VWM performance and measures of corpus callosum (CC) structure and function. Existing evidence already suggests that age-related reductions in callosal integrity are associated with poorer performance on tasks requiring the integration of information across hemispheres [24,66], although relatively few studies have examined this relationship specifically in the context of VWM. The findings reviewed above therefore provide preliminary support for the proposal that individual differences in VWM, particularly under cross-hemifield conditions, are related to variation in callosal structure and inter-hemispheric connectivity. Future work should build on these findings by directly examining whether age-related declines in VWM are mediated by changes in callosal integrity and whether these relationships are especially pronounced under conditions that place high demands on inter-hemispheric coordination. Longitudinal designs will be particularly valuable for establishing the temporal and potentially causal links between structural decline and cognitive change.

### 6.2. Experimental Manipulation of Coordination Demands

Another important direction is the systematic manipulation of inter-hemispheric coordination demands within VWM tasks. Initial evidence that age-related declines may be particularly pronounced when information must be coordinated across hemispheres rather than processed within a single hemisphere comes from the recent divided visual field one-back study discussed earlier [23]. In that study, older adults showed a disproportionately large decline in performance when successful task completion required information to be integrated across the two visual hemifields, compared with conditions in which information could be processed within a single hemifield. Although these findings are consistent with the proposal that ageing disproportionately affects inter-hemispheric coordination, they are based on a single task and therefore require further validation. Future studies should systematically assess how different levels and types of inter-hemispheric coordination demands influence VWM performance. This could be achieved by varying the spatial distribution of stimuli, the extent to which relevant information must be compared or integrated across visual fields, and the degree of integration required at retrieval. Such manipulations would allow coordination demands to be varied independently of overall memory load and attentional requirements, providing a more direct test of the proposal that age-related differences increase as demands on inter-hemispheric coordination increase.

### 6.3. Interactions with Other Mechanisms

Although the present framework emphasises inter-hemispheric coordination, this mechanism is unlikely to operate in isolation and instead interacts with processes such as attentional selection, interference control, and representational precision. Existing evidence already points to such interactions. For example, bilateral advantages are often most pronounced when attentional selection is actively engaged, and age-related declines in interference control are likely to increase the demands placed on coordination processes [53,54,55]. However, these mechanisms have rarely been examined simultaneously within a common experimental framework. A key direction for future research is therefore to determine how inter-hemispheric coordination interacts with other established determinants of VWM performance. Tasks that independently manipulate attentional load (e.g., distractor filtering), interference, and spatial distribution (unilateral versus bilateral presentation) would allow researchers to assess whether coordination demands disproportionately amplify interference or precision limitations in older adults. Such approaches would help clarify whether inter-hemispheric coordination constitutes an independent constraint on performance or whether it primarily moderates the impact of other age-sensitive mechanisms.

### 6.4. Implications for Intervention

Finally, the framework raises the possibility that interventions targeting attentional coordination and inter-hemispheric communication may help mitigate age-related declines in VWM. Existing evidence from cognitive training and non-invasive brain stimulation studies suggests that attentional control and large-scale neural connectivity can be modified [69,70,71,72], although these approaches have rarely been designed specifically to target inter-hemispheric coordination. Building on this broader literature, future research could investigate whether interventions aimed at enhancing coordination across hemispheres produce measurable benefits for VWM performance. Potential approaches include tasks designed to strengthen cross-hemifield integration, attentional training targeting spatial coordination, and non-invasive brain stimulation techniques intended to modulate inter-hemispheric connectivity. These approaches share a common goal of improving the efficiency with which distributed neural systems communicate and coordinate. Although direct evidence remains limited, the available findings suggest that coordination-based interventions represent a promising avenue for future investigation.

Together, these directions provide a framework for testing whether inter-hemispheric coordination constitutes a unifying mechanism underlying age-related decline in VWM. More specifically, several empirical predictions follow from this framework: (i) older adults should show disproportionately larger performance costs in bilateral relative to unilateral conditions when overall load is matched; (ii) performance on coordination-heavy tasks should correlate more strongly with indices of callosal integrity than performance on tasks relying primarily on intra-hemispheric processing; and (iii) training interventions targeting coordination should generalise selectively to tasks involving cross-hemifield integration, with limited transfer to tasks dominated by local processing demands.

Importantly, these predictions distinguish the present framework from accounts based primarily on local processing limitations. The coordination framework predicts that age-related deficits should increase specifically as demands on inter-hemispheric integration increase, even when memory load and attentional requirements are matched. It further predicts stronger associations between callosal integrity and performance on coordination-heavy tasks than on tasks relying primarily on intra-hemispheric processing. Conversely, the framework would be challenged if age-related deficits remained equivalent across tasks differing in coordination demands once local processing requirements were controlled, or if measures of inter-hemispheric connectivity showed little relationship with performance under conditions requiring cross-hemispheric integration.

## 7. Conclusions

Age-related decline in VWM has traditionally been attributed to reductions in storage capacity, decreases in representational precision, impairments in feature binding, and alterations in attentional control. While these accounts capture important aspects of the empirical literature, they largely assume a single, integrated processing system.

The present review has argued that this perspective is incomplete. VWM is fundamentally a distributed system, supported by lateralised neural representations that must be coordinated across the two hemispheres. Ageing is associated with structural and functional changes in the CC and associated large-scale networks, which are likely to reduce the efficiency of this coordination.

This review proposes that age-related differences in VWM reflect not only local processing limitations but also a reduced efficiency in coordinating distributed representations across hemispheres. This framework provides a unifying account linking capacity, precision, binding, and interference-based effects. By shifting the focus from local storage limitations to distributed coordination, this approach highlights the importance of network-level mechanisms in cognitive ageing. It also suggests new directions for research by emphasising how distributed neural systems are coordinated, rather than focusing solely on localised mechanisms.

Ultimately, understanding how ageing affects the coordination of distributed neural systems may be critical for developing a comprehensive account of visual working memory decline across the lifespan, and may inform interventions targeting coordination processes in ageing.

## Data Availability

No new data were created or analysed in this study.

## Abbreviations

The following abbreviations are used in this manuscript:

VWM	Visual Working Memory
CC	Corpus Callosum
CDA	Contralateral Delay Activity
IPS	Intraparietal Sulcus
DVF	Divided Visual Field
BFA	Bilateral Field Advantage
